# 'Changing the World': The Africa Regional Sexual and Gender-Based Violence Network

**DOI:** 10.1186/1753-6561-9-S4-A1

**Published:** 2015-07-07

**Authors:** Chi-Chi Undie, Harriet Birungi, Ian Askew

**Affiliations:** 1 Population Council, P.O. Box 17643-00500, Nairobi, Kenya

## Editorial

The last thirty years have been characterized by tremendous growth in the number of existing health networks world-wide; yet, little is known about why such networks emerge, what their effects are, and their roles in the governance of health [1].

The 'Africa Regional Sexual and Gender-Based Violence (SGBV) Network' emerged in the East and Southern African region in 2006. Led and coordinated by the Population Council, this network materialized as a consequence of the landscape of SGBV work in the region. At the time, while discourse and initiatives related to SGBV prevention were not unheard of, far less attention was being given to programmatic response to the service needs of SGBV survivors. With initial funding from the Swedish-Norwegian Regional HIV and AIDS Team for Africa, Embassy of Sweden, Lusaka, Zambia, the Population Council identified a number of implementing partner organizations in East and Southern Africa to facilitate collective action around the issue of responding to the needs of SGBV survivors in the region. Since then, network partner organizations have continued to work on fostering a multi-sectoral response (centered on those who have already experienced SGBV) in their countries and beyond - exploring what such a response means in low-resource settings, what it *can *mean, and what it should mean, given the constraints and realities. Each partner contributes toward the network by strengthening the capacities of the medical, legal, and/or justice sectors to care for survivors of SGBV, and by building an evidence base for SGBV programming. Partners develop, implement, and evaluate core elements of a multi-sectoral response model that incorporates the overlapping and complementary responsibilities of the health, police and justice, and social service sectors. The ethos of the *Africa Regional SGBV Network *centers on the conviction that survivors require access to all services, but that it may not be feasible, appropriate, or cost-effective to deliver all services in one location. Through their interventions over the years, network partners have not taken the conventional understanding of a multi-sectoral approach for granted, but have instead allowed other viewpoints and actions to emerge in regard to such an approach. Where necessary, partners have conceived programmatic response to survivors' service needs differently, testing out innovative and, sometimes, daring approaches.

On December 4, 2013, for the first time ever, the *Africa Regional SGBV Network *convened a meeting in Washington, DC, USA. The meeting commemorated the *16 Days of Activism Against Gender Violence *and raised awareness of interventions for survivors of SGBV in low-resource contexts. The forum reflected a culmination of efforts from seven years of grounded research and practice-building in the field of SGBV, and brought together network partner organizations from Kenya, South Africa, Swaziland, and Zambia, in addition to policy, practitioner, and researcher audiences from the Washington, DC area.

This volume of extended abstracts summarizes network partner presentations delivered during the meeting. The presentations centered on each partner's interventions and main results, coupled with the effectiveness of partners' endeavors in responding to the needs of survivors. The notion of 'effectiveness' as employed here draws on Shiffman's [1] definition of the concept in regard to networks and their policy consequences. As he explains:

Effectiveness refers to the extent to which networks are able to *change the world to meet their members' perceptions of what reality should look like *[2]. We examine effectiveness by considering ... policy consequences ... Policy consequences pertain to the global policy process, including international resolutions, funding, national policy adoption, and the scale-up of interventions (emphasis added).

Accordingly, this collection of extended abstracts highlights the nature of policy consequences that the efforts of the *Africa Regional SGBV Network *have engendered in the East and Southern African region.

The current volume appears during an exciting time of 'climate change' in SGBV work, where SGBV prevention initiatives in particular are receiving mounting attention in the East and Southern African region. While there is good reason for this near-singular focus on prevention, this volume will remind readers that any supposed dichotomy between 'prevention' and 'response' is artificial, at best. Both are closely interconnected and critical, and work hand in hand with one another. In responding to SGBV, we prevent it - and in preventing SGBV, we respond to it. Furthermore, compared to other parts of the world, SGBV 'prevention' and 'response' alike are in their infancy in East and Southern Africa.

In responding to SGBV, and in bringing about policy consequences (as defined above), the Population Council and its partners are changing the way the world thinks about the critical health and development issue that SGBV represents in East and Southern Africa. Indeed, as each of the extended abstracts in this volume demonstrates, in diverse ways, the *Africa Regional SGBV Network *is 'changing the world' to meet its members' perceptions of what reality should look like in regard to programmatic response to the needs of survivors in the region.

## References

[B1] Shiffman J The emergence and effectiveness of global health networks Population Reference Bureau 2013, December http://www.prb.org/Publications/Articles/2013/shiffman-global-health-networks.aspx Interviewer: H. Worley

[B2] Sikkink K Miles Kahler Networked Politics: Agency, Power, and Governance 2009 Ithaca, NY: Cornell University Press

